# Closed Shell Iron(IV) Oxo Complex with an Fe–O Triple Bond: Computational Design, Synthesis, and Reactivity

**DOI:** 10.1002/anie.202009347

**Published:** 2020-10-29

**Authors:** Erik Andris, Koen Segers, Jaya Mehara, Lubomír Rulíšek, Jana Roithová

**Affiliations:** ^1^ Institute for Molecules and Materials Radboud University Heyendaalseweg 135 6525 AJ Nijmegen The Netherlands; ^2^ Institute of Organic Chemistry and Biochemistry of the Czech Academy of Sciences Flemingovo náměstí 2 16610 Praha 6 Czech Republic

**Keywords:** ion spectroscopy, iron oxo complexes, ligand design, spin state

## Abstract

Iron(IV)‐oxo intermediates in nature contain two unpaired electrons in the Fe–O antibonding orbitals, which are thought to contribute to their high reactivity. To challenge this hypothesis, we designed and synthesized closed‐shell singlet iron(IV) oxo complex [(quinisox)Fe(O)]^+^ (**1^+^**; **quinisox‐H**=(*N*‐(2‐(2‐isoxazoline‐3‐yl)phenyl)quinoline‐8‐carboxamide). We identified the quinisox ligand by DFT computational screening out of over 450 candidates. After the ligand synthesis, we detected **1^+^** in the gas phase and confirmed its spin state by visible and infrared photodissociation spectroscopy (IRPD). The Fe–O stretching frequency in **1^+^** is 960.5 cm^−1^, consistent with an Fe–O triple bond, which was also confirmed by multireference calculations. The unprecedented bond strength is accompanied by high gas‐phase reactivity of **1^+^** in oxygen atom transfer (OAT) and in proton‐coupled electron transfer reactions. This challenges the current view of the spin‐state driven reactivity of the Fe–O complexes.

## Graphical abstract

An iron oxo complex with an Fe≡O bond and a closed shell ground state was designed by employing DFT calculations, synthesized, and experimentally characterized by gas‐phase spectroscopic techniques. Despite the unprecedented Fe≡O bond strength and closed‐shell ground state, it still exhibits high‐reactivity.

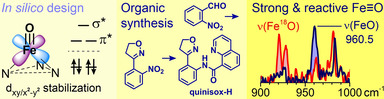

## Introduction

Iron(IV)‐oxo units act as powerful oxidants in many enzymatic systems.[^1^, ^2^, ^3^, ^4^, ^5^] The scope of their reactivity includes reactions such as hydrogen atom transfer (HAT)^[6]^ or electrophilic attacks to arene rings.[^7^, ^8^] It has been well established that the reactivity correlates with the spin state^[9]^ that may vary along the reaction coordinate (multi‐state reactivity).^[10]^ In particular, HAT reactions in non‐heme iron(IV)‐oxo complexes have been generally predicted to proceed via high spin transition states,[^11^, ^12^, ^13^] irrespective of the spin state of the initial iron‐oxo complexes, a phenomenon known as two‐state reactivity.[^14^, ^15^] The HAT reactions are frequently of a radical character,^[16]^ but closed‐shell oxidants can initiate them as well,[^17^, ^18^] for example via proton‐coupled electron transfer.^[19]^ In this respect, reactivity of so far unknown closed shell iron‐oxo complexes can elucidate the role of unpaired electrons in Fe‐O mediated oxidations.[^20^, ^21^]

**Figure 1:**
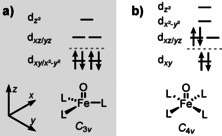
Electronic configurations of singlet d^4^ iron oxo complexes in a) *C*_3*v*_ and b) *C*_4*v*_ symmetries. The dashed lines separate orbitals with Fe‐O non‐bonding and Fe‐O antibonding character.

Stabilization of low‐spin iron(IV)‐oxo compounds can be best achieved, by analogy with iron(IV) nitrides,^[22]^ in (pseudo) *C*_3*v*_ symmetric environment (Figure 1). This promotes (near) degeneracy of the d_*xy*_/$d_{x^{2}-y^{2}}$ orbitals and their stabilization with respect to remaining three d orbitals, resulting in an electronic configuration with four d electrons in essentially non‐bonding orbitals.^[23]^ We note in passing that Kojima achieved stabilization of singlet d^4^ ruthenium(IV)‐oxo complexes also in (pseudo) *C*_5*v*_ symmetric environment.^[24]^ The expected electronic configuration corresponding to *S*=0 in *C*_3*v*_ symmetric environment would then suggest the Fe‐O bond order of 3. The Fe‐O bond would be considerably stronger than so far observed iron‐oxo species and shall appear in the “fingerprint” of an IR spectrum. Even though no compounds with triple Fe‐O bonds have been identified yet, other Fe‐X triple bonds exist. There are well‐characterized complexes with nitride ligands.[^22^, ^23^, ^25^, ^26^, ^27^] Fe‐X triple bonds also exist with carbyne ligands^[28]^ and mass‐spectrometry has suggested existence of triple bonds with heavier group 14 and 15 elements,[^29^, ^30^] or even the existence of the Fe‐B quadruple bond.^[31]^ However, these bonds have not been characterized by means of vibrational spectroscopy.

## Results and Discussion

**Figure 2:**
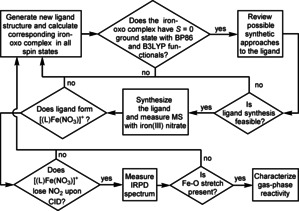
Chart describing the workflow in ligand selection process.

**Computer‐Aided Iterative Ligand Design**. Our strategy, outlined in Figure 2, was to identify ligands favoring formation of *S*=0 [(L)Fe^IV^(O)]^+^ ground state. This involved “DFT screening” of [(L)Fe(O)]^+^ complexes, synthesis of the most promising ligands, preparation of the corresponding [(L)Fe^III^(NO_3_)]^+^ complexes, their transfer to the gas phase and their fragmentation to form [(L)Fe^IV^(O)]^+^ by a procedure demonstrated previously.[^32^, ^33^] To verify the *S*=0 [(L)Fe^IV^(O)]^+^ ground state, we employed gas‐phase IR/vis ion spectroscopy.[^34^, ^35^, ^36^, ^37^, ^38^, ^39^, ^40^, ^41^, ^42^]

We thus created a library of candidate structures (more than 450 ligands; *cf*. Supporting Information) and optimized their geometries employing the computationally affordable BP86‐D3 functional (owing to efficient density‐fitting approximation) and the def2‐SVP basis set in all spin states. Promising structures were then recalculated by the B3LYP‐D3 functional, which is known to favor higher spin states owing to its 20 % fraction of Hartree–Fock exchange.[^43^, ^44^] Our goal was to obtain a set of ligands that would form [(L)Fe^IV^(O)]^+^ in the *S*=0 ground state favored by more than 2 kcal mol^−1^, predicted by both functionals (which is, in fact, always limited by the B3LYP‐D3 prediction) and that would be synthetically accessible. For the sake of the clarity of further discussion, relative energies calculated at B3LYP‐D3/6–311+G** [*E* ^0K^_s_, *E* ^0K^_t_, *E* ^0K^_q_] are displayed next to each of the ligands mentioned below. In cases, where we terminated calculations at the pre‐screening stage, we state only the BP86‐D3/def2‐SVP energies. At the BP86‐D3 level, the triplet and quintet are destabilized, on average, by 6.9 and 15.1 kcal mol^−1^, respectively, compared to the B3LYP‐D3 energies (Figure S1 in the Supporting Information).

**Figure 3:**
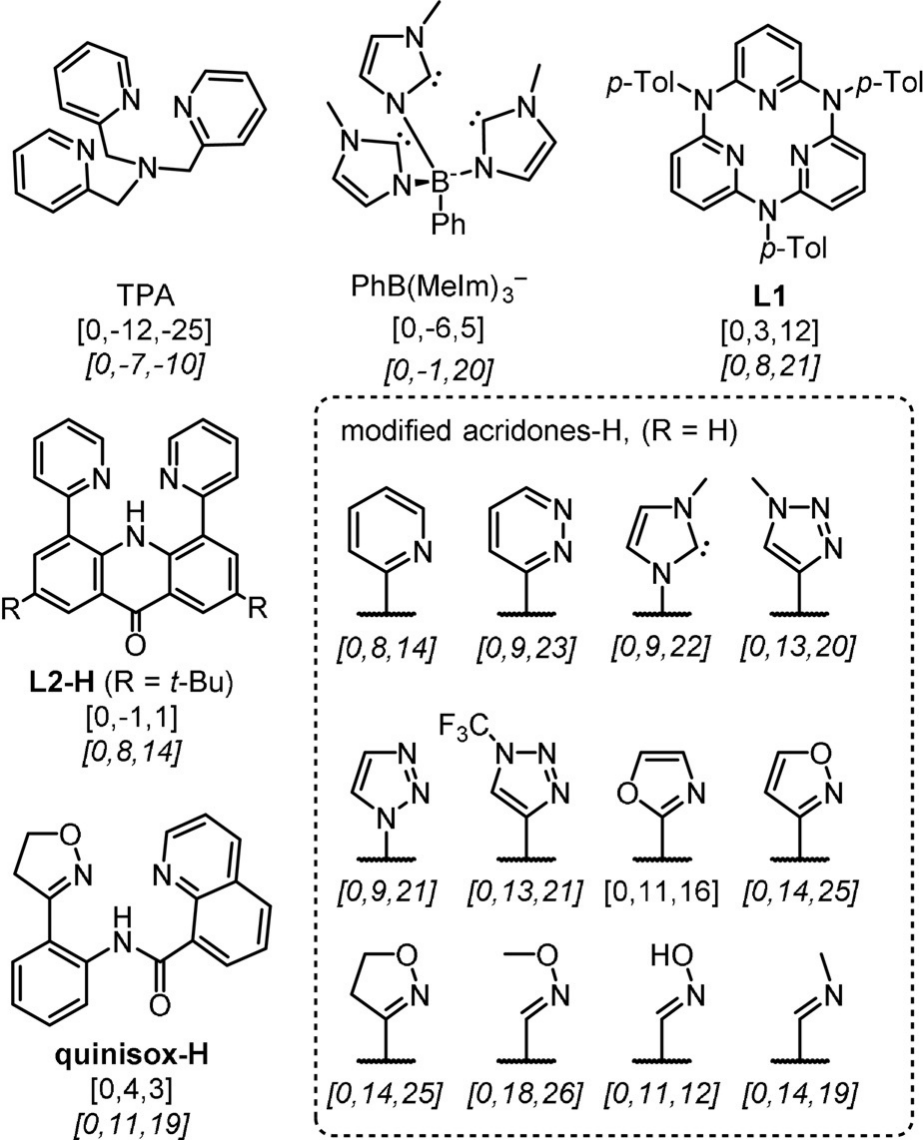
Some of the ligands considered for the formation of the *S*=0 iron(IV) oxo complexes. The full list can be found in the Supporting Information. The values in the square brackets are relative energies of the singlet, triplet and quintet states with respect to the singlet state at 0 K calculated at the B3LYP‐D3/6–311+G** level. BP86‐D3/def2‐SVP values are given in italics. Note that at the BP86‐D3 level, the triplet and quintet are destabilized, on average, by 6.9 and 15.1 kcal mol^−1^, respectively, compared to the B3LYP‐D3 level (Figure S1 in the Supporting Information).

The singlet state for the commonly employed TPA ligand [0, −12, −25 kcal mol^−1^] is very high in energy (Figure 3). The situation is somewhat more favorable for the *N*‐heterocyclic carbene‐based ligand PhB(Im)_3_^−^ [0, −6, 5 kcal mol^−1^], which served as our model for ligands that stabilize *S*=0 state in iron(IV) nitrides.^[22]^ However, our computations predict that the triplet state is still considerably lower in energy than the singlet state. After initial rounds of screening we found that ligands with pyridine rings, which form a plane perpendicular to the Fe‐O unit seemed to have the most stable *S*=0 states. We hypothesize, that this orientation minimizes the interaction between ligand orbitals and the Fe‐O unit's d_*xy*_ and $d_{x^{2}-y^{2}}$ orbitals. This orientation was achieved with rigid sp^2^‐bound ligand frameworks, like azacalix[3](2,6)pyridine‐based ligand **L1** [0, 3, 12 kcal mol^−1^].^[45]^ Unfortunately, upon mixing the neutral ligand with iron(III) nitrate, we did not observe any formation of an iron(III) complex, but only the protonated ligand [(**L1**)H]^+^ appeared in the mass spectrum (Figure S2 in the Supporting Information). To make the ligand less rigid and thus to facilitate accommodation of the iron center, we considered modification of existing acridine‐based dtdpa (2,7‐di‐*tert*‐butyl‐4,5‐di(pyridin‐2‐yl)acridone) ligand^[46]^ to acridone **L2‐H** (Figure 3). We have generated [(**L2**)Fe^III^(NO_3_)]^+^ which lost NO_2_ upon collisional activation (Figure S3 in the Supporting Information). However, the resulting complex was unreactive and its IRPD spectrum did not show any Fe‐O stretching (ν(Fe‐O)) vibration (Figure S4 in the Supporting Information). We hypothesized that aromatic hydroxylation of one of the pyridines might have occurred,^[47]^ possibly via a roll‐over mechanism.[^48^, ^49^] To enhance the stability of the singlet spin state and to prevent the ligand oxidation, we performed screening of functional groups that could substitute the pyridine rings at the acridone skeleton. (Figure 3, dashed box; all structures are in the Supporting information). This screening uncovered a simple methyl oxime as best substituent. However, methyl oxime is quite flexible and its N−O bond could thus get cleaved during the collisional activation of nitrate. Therefore, we went with the second best group—isoxazoline—which should be conformationally rigid and has only aliphatic C−H bonds, which are more difficult to oxidize.

Because attaching the isoxazoline unit to acridone skeleton was not compatible with the synthesis we used for the acridone, we considered breaking the middle ring of the acridone to offer quinoline 8‐carboxylic acid as a building block. This led to the design of the **quinisox‐H** ligand that was synthetically feasible and the singlet spin state of [(quinisox)Fe^IV^(O)]^+^ (**1^+^**) was computed to be >3 kcal mol^−1^ more stable than the triplet and quintet states.

**Multireference Quantum Chemical Calculations**. In‐depth analysis of the Fe‐O bond in **1^+^** which may also *a posteriori* validate (or calibrate) used DFT methodology requires multi‐reference complete‐active space self‐consistent field (CASSCF) calculations. Active space in our state‐specific CASSCF calculations comprised five Fe‐centered molecular orbitals (originating in iron *d* orbitals) and three *p* orbitals on oxygen atoms (Figure S5). The weight of the dominant electronic configuration for the singlet, triplet, and quintet states were 84, 82 %, and 79 % respectively, which provides reasonable justification for usage of single‐reference DFT methods. The dominant electronic configuration for the singlet, in agreement with the DFT results, is (d_*xy*_)^2^ ${\left(d_{x^{2}-y^{2}}\right)}^{2}$ (π_*x*_)^2^ (π_*y*_)^2^ (σ_*z*_)^2^ (π_x_*)^0^ (π_y_*)^0^ (σ_z_*)^0^, which corresponds to the Fe≡O electronic structure. Natural bond order analysis also shows that the Fe‐O bond order is 2.82.

**Gas‐Phase Preparation of 1^+^**. We added ≈0.5 mg of **quinisox‐H** and ≈0.5 mg of Fe(NO_3_)_3_⋅9 H_2_O in 2 mL acetonitrile and sprayed the supernatant solution. Corresponding ESI‐MS spectrum shows the ion at *m*/*z* 434, which corresponds to [(quinisox)Fe(NO_3_)]^+^ ions (Figure S6a in the Supporting Information). These ions lose NO_2_ molecules in collision‐induced dissociation with xenon,^[33]^ forming **1^+^** (*m*/*z* 388) (Figure S6b in the Supporting Information). Typical ionization conditions to generate **1^+^** were: 3–6 kV spray voltage, 250 °C capillary temperature, 20–35 V capillary voltage 115–160 V tube lens voltage, 5 psi sheath gas pressure, 20–45 a.u. auxiliary gas flow.

**Figure 4:**
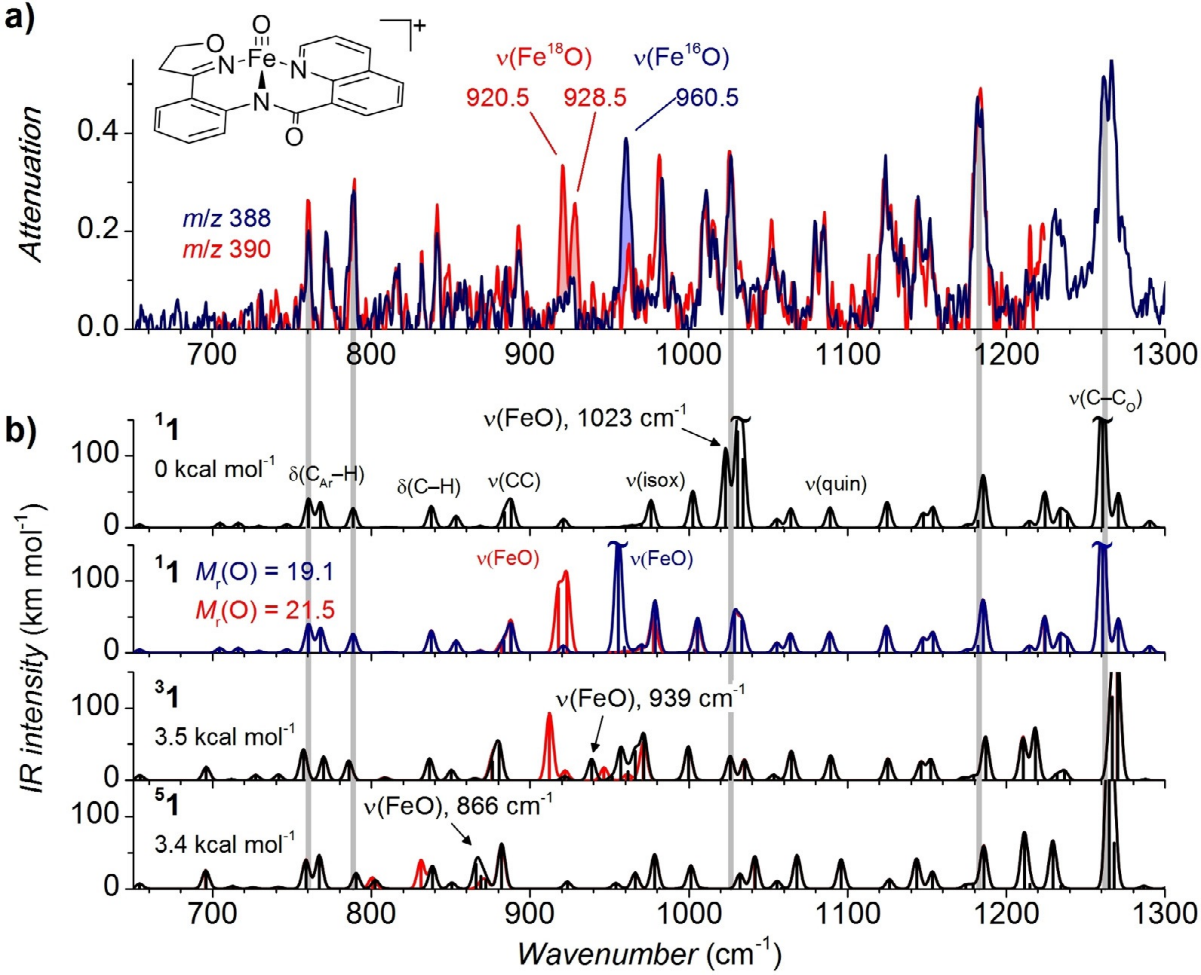
a) Neon tagging IRPD spectrum of **1^+^** (blue trace) and **1^+^‐^18^O** (red trace). b) Theoretical IR spectra for the singlet (top panel), singlet with the shifted Fe‐O band (second panel; mass of the O atom is indicated in the Figure), triplet (third panel) and quintet (bottom panel) calculated with B3LYP‐D3/6–311+G** level of theory. Predictions for the Fe‐^18^O‐labeled isomers are shown in red. Theoretical vibrational frequencies were scaled by 0.98. Indicated relative energies correspond to enthalpies at 0 K.

**IRPD Spectra: Experiment and Theory**. To prove that **1^+^** indeed corresponds to the iron‐oxo compound, we acquired neon tagging IRPD spectra in the Fe‐O band frequency range (Figure 4 a, blue trace). The spectra were measured by introducing the measured ions (**1^+^**) in a cryogenic ion trap, where they form weakly‐bound complexes with neon atoms. Wavenumber‐dependent dissociation of these neon complexes with tunable IR laser provides the IRPD spectrum (more details can be found in the Supporting Information).

We have previously shown that ν(Fe‐O) vibrational frequencies in the gas phase are blue shifted by ≈10 cm^−1^, compared to their values in solution. Thus, the values reported herein are directly comparable with the literature values reported mostly for the species in the condensed phase.^[38]^ To determine the ν(Fe‐O) band position, we carried out ^18^O isotopic labeling; **1^+^‐^18^O** (*m*/*z* 390) was prepared by adding 6 equivalents of HN^18^O_3_ to the sprayed solution (Figure 4 a, red trace).

Comparison of the two spectra in Figure 4 a shows that a single band at 960.5 cm^−1^, which we assign to the Fe‐O stretching vibration, disappears upon the ^18^O labeling and a Fermi doublet comprising of two new bands—stronger at 920.5 cm^−1^ and weaker at 928.5 cm^−1^—appears instead. The presence of the Fermi doublet can be explained by vibrational coupling of the shifted ν(Fe‐O) band with the weak band at 926 cm^−1^ in the spectrum of **1^+^**. Since the 926 cm^−1^ band is weaker than the 960.5 cm^−1^ band, the greater intensity of the 920.5 cm^−1^ band as compared to the 928.5 cm^−1^ band suggest that the band at 920.5 cm^−1^ indeed carries the most of the Fe‐O stretching character. The ^18^O labeling shift is thus −40 cm^−1^, which is fully consistent with the value of −42 cm^−1^ predicted by a simple harmonic oscillator model for Fe‐O diatomic.

Further support for these assignments comes from the DFT calculations. We calculated theoretical IR spectra (B3LYP‐D3/6–311+G**) for all spin states of **1^+^**. In our experience, B3LYP‐D3/6–311+G** provides the best predictions for ligand vibrations,[^50^, ^51^] while it significantly blue‐shifts Fe‐O stretching bands by 50–100 cm^−1^ in iron(IV)‐oxo complexes.[^38^, ^52^] Majority of the bands in these spectra correspond to the C−H bending modes and the skeletal stretching modes and show a good agreement with the measured IR spectra (Figure 4 b). As can be seen, the main difference among the calculated IR spectra for the different spin states of **1^+^** is the position of the ν(Fe‐O) band (1023, 939, and 866 cm^−1^ for ^**1**^ **1^+^**, ^**3**^ **1^+^**, and ^**5**^ **1^+^**, respectively). Considering the abovementioned typical shift in Fe‐O frequencies at the B3LYP level, the computed value for the singlet state (${\tilde{\nu }}_{\mathrm{predicted}}=$ 1023 cm^−1^) seems reasonable. Our calculations can also reproduce the Fermi doublet arising from the coupling between the Fe‐^18^O stretching band and the ligand band at 926 cm^−1^. To compensate for the incorrect prediction of the ν(Fe‐O) force constant, we increased the mass of the O atom in ^**1**^ **1^+^** to 19.1, which moved the ν(Fe‐O) vibration to the correct position^[53]^ of ≈960 cm^−1^ (Figure 4 b, second panel, red trace). Then increasing this mass by the 18/16 ratio to a value of 21.5, we can clearly observe the Fermi doublet like in the IRPD experiment (compare Figure 4 a, red trace and Figure 4 b, second panel, red trace).

The Fe‐O stretching frequencies in the reported iron(IV) and iron(V)‐oxo complexes lie between 799 and 862 cm^−1^.[^54^, ^55^, ^56^] The highest Fe‐O stretching frequency so far (885 cm^−1^) has been assigned for the iron(IV)‐oxo unit inside a zeolite framework.^[57]^ Thus, the Fe‐O vibration in our complex is blue‐shifted by more than 100 cm^−1^ with respect to the typical iron(IV)‐oxo stretching vibrations (and by 75 cm^−1^ with respect to the Fe‐O unit in the zeolite). In fact, this value is much closer to the frequencies of the Fe≡N triple bonds found in the singlet iron(IV)‐nitrido complexes.^[22]^ For example, the Fe‐N stretching frequency in [(TIMEN^Xyl^)Fe(N)] is 1008 cm^−1^. Scaling this frequency using harmonic oscillator model for substitution of nitrogen by oxygen results in a value of 956 cm^−1^, which is almost the same frequency as determined for **1^+^**. Similar stretching frequencies are also typical for metal‐oxo complexes with triple M≡O bonds such as d^2^ manganese(V) oxo complexes.[^58^, ^59^]

The Fe‐O stretching frequency also allows us to estimate the Fe‐O distance. Employing Badger's rule^[60]^ with parameters tuned for the heme iron systems,^[61]^ the estimated *d*(Fe‐O) is 1.58 Å. Using our recent linear correlation,^[62]^ the estimate would be 1.48 Å. The B3LYP‐D3/6–311+G** Fe‐O bond distance if 1.54 Å, in between the two estimates. All of these estimates of the Fe‐O bond length are comparable or smaller than in the low‐spin d^3^ [Fe^V^(O)(TAML)]^−^ complex.^[63]^

**Figure 5:**
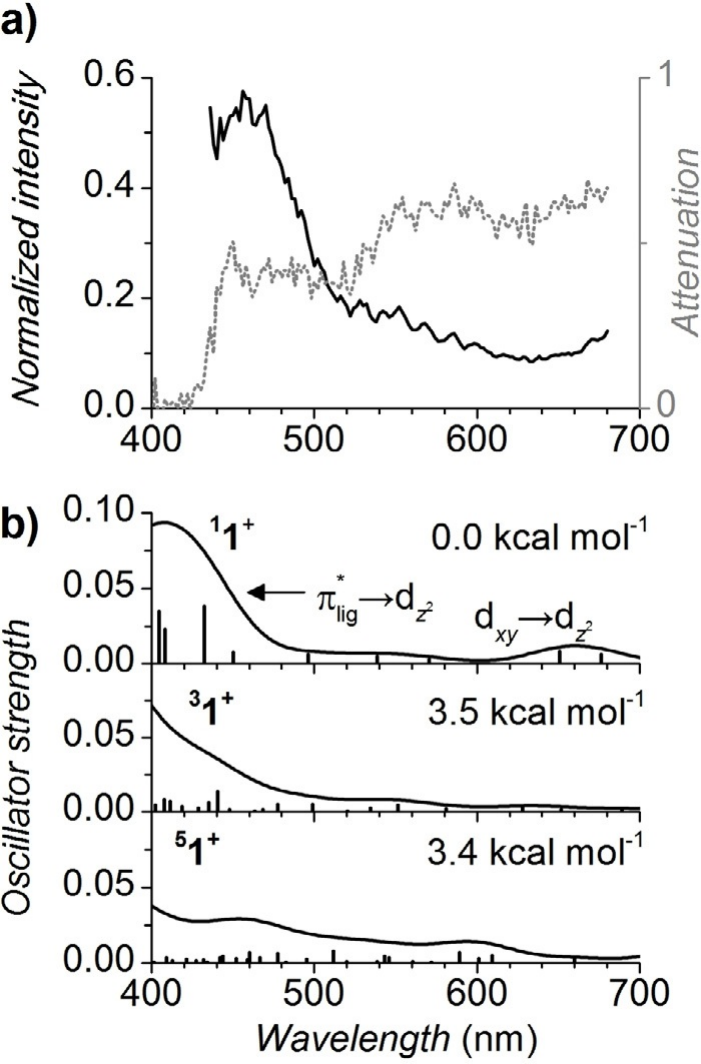
a) Neon tagging VIS photodissociation spectrum of **1^+^**: the attenuation (1−*N*/*N*_0_; grey dashed trace) and the intensity normalized by the laser power (−ln(*N*/*N*_0_)/*P*_laser_; black trace). b) TD‐DFT predictions of VIS spectra of **1^+^** in the singlet, triplet, and quintet states. The relative energies refer to enthalpies at 0 K.

**Visible Photodissociation Spectra**. Next, we measured the visible neon tagging photodissociation spectrum in the 430–680 nm region. (Figure 5 a). The spectrum shows a band at 460 nm with a small shoulder at 560 nm and an onset of a band at 680 nm (our setup does not allow to measure longer wavelengths). Overall, the shape of this spectrum matches the computed spectrum for the singlet spin state quite well (Figure 5 b, top panel). Accordingly, the 680 nm band corresponds to the $d_{xy}\to d_{z^{2}}$ transition and the 430 nm band corresponds to the $\pi {}^{*}\to d_{z^{2}}$ transitions, where π* are the antibonding orbitals from the oxime and the quinoline parts of the ligand.

**Figure 6:**
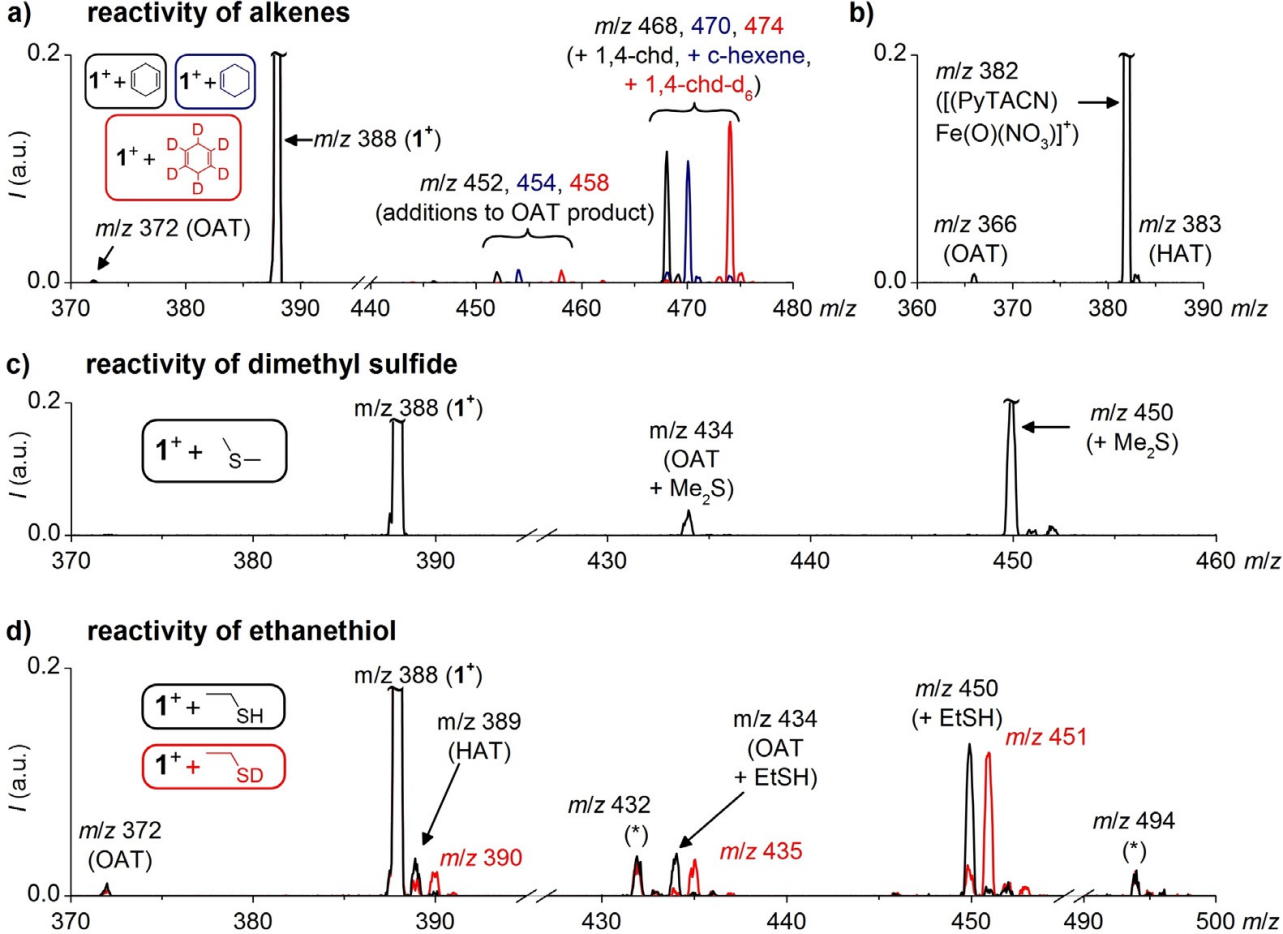
Reactivity of **1^+^** in the gas phase at nominally zero collision energy with a) 1,4‐cyclohexadiene (*p*=0.17 mTorr, black trace), 1,4‐cyclohexadiene‐1,2,3,4,5,6‐d_6_ (*p*=0.20 mTorr, red trace), and cyclohexene (*p*=0.23 mTorr, blue trace). b) Reactivity of [(PyTACN)Fe(O)(NO_3_)]^+^ (**3^+^**) with 1,4‐cyclohexadiene (*p*=0.19 mTorr);^[32]^ **3^+^** was generated by oxidation of [(PyTACN)Fe(OTf)_2_] with AcOOH in the presence of HNO_3_ in acetonitrile. c) Reactivity of **1^+^** with dimethyl sulfide (*p*=0.19 mTorr) and d) with ethanethiol (*p*=0.19 mTorr, black trace) and ethane(thiol‐d) (*p*=0.19 mTorr, red trace, peaks marked with asterisks are discussed in the Supporting Information).

**Gas Phase Reactivity**. Finally, we explored gas‐phase reactivity of the singlet spin state **1^+^** complex. Hallmark reactivity of iron(IV)‐oxo complexes involves activation of strong C−H bonds. However, **1^+^** did not react with cyclohexane, nor with benzene. It did react with 1,4‐cyclohexadiene (CHD), that is, reactant with weaker C−H bonds; however, with a different reactivity pattern than all previously reported singly charged iron(IV)‐oxo complexes. Generally, the complexes dominantly yielded HAT reactions alongside minor OAT reactions.^[38]^ Our singlet complex **1^+^** does not abstract a hydrogen atom from 1,4‐cyclohexadiene, but reacts very efficiently by the addition reaction (*m*/*z* 468) and by the OAT reaction followed by addition of the alkene molecule (*m*/*z* 450, Figure 6 a). We assume that the addition involves epoxidation of the CHD with the Fe≡O unit. We deem the alternative bare coordination as unlikely, because we did not observe any coordination in the reaction of **1^+^** with benzene and in the reaction of related [(quinisox)Fe(OMe)]^+^ with CHD (see Figure S7 in the Supporting Information). In addition, collision‐induced dissociation of the adduct (Figure S8a in the Supporting Information) leads dominantly to the loss of oxygenated cyclohexadiene fully consistent with the reactive coupling. We also excluded the alternative reaction pathway leading to the hydroxylation of the C−H bond by the abstraction/rebound mechanism. Complex **1^+^** reacts in the same way also with cyclohexene and *d*_6_‐labeled cyclohexadiene. Deuteration of CHD does not result in any apparent kinetic isotope effect which rules out the involvement of C−H bonds in the rate determining step.

For comparison with other iron(IV)‐oxo complexes, we measured the reactivity of previously reported [(PyTACN)Fe(O)(NO_3_)]^+^ (**3^+^**). Complex **3^+^** has been generated by oxidation in solution which yields preferentially the *S*=1 complex. Under the identical reaction conditions, **3^+^** reacts with CHD by HAT and OAT in a roughly 1:1 ratio (Figure 6 b). Therefore, **1^+^** is highly selective for the addition reaction (epoxidation), in comparison with **3^+^**. Also, **1^+^** is an order of magnitude more reactive than **3^+^**, which is a very high reactivity, compared to other iron(IV)‐oxo complexes in the gas phase.^[38]^

We have further confirmed the large reactivity of **1^+^** in oxygen transfer reactions with dimethyl sulfide and with ethanethiol. Dimethyl sulfide reacts in analogy as CHD: the addition reaction (*m*/*z* 450), OAT (*m*/*z* 372, very small intensity), and OAT followed by the association reaction (*m*/*z* 434) (Figure 6 c). Ethanethiol also shows the same reactivity pathways corresponding to the oxygenation reactions (Figure 6 d): the addition reaction (*m*/*z* 450), OAT (*m*/*z* 372), OAT followed by the association with ethanethiol (*m*/*z* 434). In addition, we also observe the HAT reaction (*m*/*z* 389). This reactivity is most likely due to proton coupled electron transfer reactivity as we observed previously for iron(III)‐oxo complexes.^[62]^ The assignment of the reaction channels is further confirmed by control experiments with EtSD.

## Conclusion

In this work, we successfully applied iterative computer‐aided ligand design employing (relatively fast) DFT calculations to identify potential candidate singlet iron(IV)‐oxo complexes. The most promising candidates were then synthesized and characterized by gas‐phase vibrational and electronic spectroscopies and correlated with DFT and multi‐reference wave‐function quantum chemical calculations. In particular, [(quinisox)Fe(O)]^+^ (**1^+^**) complex is the first *S*=0 Fe^IV^‐oxo complex characterized up to date. The IRPD spectroscopy revealed the presence of very strong Fe‐O bond in **1** ^+^ ($\tilde{\nu }=$ 960.5 cm^−1^), which is fully consistent with the Fe≡O triple bond predicted by theory. Interestingly, despite the presence of the strong Fe‐O bond and the absence of the spin density on the oxygen atom, complex **1^+^** is still highly reactive and shows unusual selectivity for epoxidation (as compared to the C−H bond activation) in alkenes. To what extent is this reactivity a unique feature of singlet iron(IV)oxo complexes or a side‐product of the ligand field required to stabilize the singlet state remains an open question, which could be solved by theoretical studies.

## Conflict of interest

The authors declare no conflict of interest.

## Supporting information

As a service to our authors and readers, this journal provides supporting information supplied by the authors. Such materials are peer reviewed and may be re‐organized for online delivery, but are not copy‐edited or typeset. Technical support issues arising from supporting information (other than missing files) should be addressed to the authors.

Supplementary

Supplementary

Supplementary

Supplementary
