# Supplementary material for: Closed Shell Iron(IV) Oxo Complex with an Fe–O Triple Bond: Computational Design, Synthesis, and Reactivity
Source: Angew Chem Int Ed Engl. 2020 Oct 29;59(51):23137–44. doi: 10.1002/anie.202009347 (PMC7756500; doi:10.1002/anie.202009347)
Supplement: Supplementary file 4 — Supplementary [file ANIE-59-23137-s004.pdf]

Author Contributions

E.A. carried out the computational screening and analysis. E.A. and K.S. performed the organic synthesis, E.A., K.S. and J.M. performed mass-spectrometry measurements. E.A. performed ion spectroscopy measurements. E.A., L.R. and J.R. wrote the manuscript. All authors commented on the manuscript.
